# Editorial: Investigating the impact of bioactive metabolites and extracts in human health and disease

**DOI:** 10.3389/fmolb.2023.1244316

**Published:** 2023-08-07

**Authors:** Sanjeev Kumar, Vibhav Gautam, Bhim Pratap Singh, Deepak Kumar

**Affiliations:** ^1^ Department of Biochemistry and Molecular Biology, Michigan State University, East Lansing, Michigan, United States; ^2^ Centre of Experimental Medicine and Surgery, Institute of Medical Sciences, Banaras Hindu University, Varanasi, Uttar Pradesh, India; ^3^ Department of Agriculture and Environmental Sciences (AES), National Institute of Food Technology Entrepreneurship and Management (NIFTEM), Sonepat, Haryana, India; ^4^ Department of Botany, Institute of Science, Banaras Hindu University, Varanasi, Uttar Pradesh, India

**Keywords:** bioactive metabolites, human health, ethnopharmacology, plant extracts, medicinal and aromatic plants

## Introduction

Plants have long served as valuable sources of potent and diverse compounds with multiple medicinal properties. Across various cultures and regions, traditional medicine has relied on plant-based remedies for centuries. Phytochemicals, found in plants, often exhibit a range of medicinal properties, including cytotoxic, ant proliferative, antioxidant, anti-aging, and therapeutic effects on metabolic disorders. In comparison to synthetic drugs, treatments based on phytochemicals offer several advantages, such as enhanced safety profiles, eco-friendliness, and cost-effectiveness (Newman and Cragg, 2020). Given the immense medicinal importance of plant products and their metabolites, this proposed Research Topic aims to explore the impact of bioactive metabolites and extracts on human health and disease. By delving into the research surrounding these natural compounds, scientists and researchers can uncover valuable insights into their mechanisms of action, therapeutic potential, and their application in various health conditions (Newman and Cragg, 2020). This exploration contributes to a deeper understanding of the potential benefits of plant-based treatments and their metabolites, paving the way for the development of new therapeutic strategies, improving human health outcomes, and addressing the challenges posed by different diseases (Olivares-Vicente et al., 2018).

The Research Topic of four articles presents compelling research on the medicinal importance of various phytochemicals derived from different plants. The data presented in these articles is particularly intriguing, highlighting the potential of plant metabolites and their extracts in the treatment of neurological disorders such as Alzheimer’s disease, cancer, and diabetes mellitus. The findings provide valuable insights into the therapeutic properties of these phytochemicals and their potential applications in tackling these challenging health conditions. The research conducted in these articles offers promising avenues for the development of novel treatment approaches and interventions using natural compounds derived from plants. By exploring the medicinal potential of phytochemicals, researchers are expanding our understanding of their mechanisms of action and their ability to target specific disease pathways. This knowledge opens up new possibilities for the development of effective and safe therapies that can contribute to the management and treatment of Alzheimer’s disease, cancer, and diabetes mellitus. The data presented in these articles shed light on the potential of plant-based medicine as a valuable resource in the fight against these serious health conditions, providing hope for future advancements in treatment and improved patient outcomes Qin et al.; Hafeez et al.; Sahu et al.).

The first article, as reported by Qin et al. explored the medicinal importance of the plant *Ligusticum chuanxiong* Hort (CX). By using the leaves and rhizome extract of the CX plant authors detected more than 70 compounds which mainly includes phthalides, alkaloids, organic acids, terpenes, and polyphenols. Through the utilization of these extracts, the authors made a discovery that CXs exhibit protective effects against oxidative stress. Specifically, they found that CXs have the ability to prolong the lifespan of *C. elegans* N2, alleviate lipofuscin, malondialdehyde (MDA), and reactive oxygen species (ROS) accumulation, improve movement levels, and enhance antioxidant enzyme activity. These findings suggest the potential of CXs as therapeutic agents with antioxidant and anti-aging properties in the model organism *C. elegans* N2. Interestingly CXR reduced the *β*-amyloid peptide (Aβ)-induced paralysis phenotype by reducing the production of Aβ in strain CL4176s and alleviated chemosensory behavior dysfunction in CL2355s (Qin et al.).

The observed reduction of Aβ (amyloid-beta) indicates that in the future, the bioactive compounds derived from CX plants may be promising novel therapeutics for the treatment of Alzheimer’s disease. However, it is important to note that further research is necessary to identify the specific bioactive molecules present in CX plants that directly contribute to the reduction of Aβ. This identification is crucial for a better understanding of the underlying mechanisms and for the development of targeted therapeutic interventions for Alzheimer’s disease. The next article, as reported by Qi et al. examines the anti-cancer property of Gypenosides (GYP). GYP is the main bioactive compound of the plant *Gynostemma pentaphyllum*, which has many medicinal properties such as lowering blood lipids and blood glucose as well as exhibiting cytotoxic and anti-tumor behavior. A serum metabolomics profiling study of mice treated with GYP unraveled the altered level of 53 metabolites. The GYP has been found to inhibit tumor growth and reduce elevated pathological symptoms in tumor-bearing mice. Additionally, it has shown the ability to restore the altered levels of 23 lung cancer biomarkers. These findings indicate the potential of GYP treatment as a therapeutic approach for lung cancer, as it not only hinders tumor progression but also mitigates associated pathological symptoms and restores the disrupted biomarker profile. Further research is warranted to fully understand the underlying mechanisms and to evaluate the translational potential of GYP treatment in lung cancer patients. Based on STRING-based analysis, it has been suggested that STAT3, MAPK14, EGFR, and TYMS are potential targets of GYP in lung cancer. These proteins are likely involved in the molecular pathways affected by GYP treatment. Furthermore, GYP has been shown to enhance the efficacy of the anticancer drug cisplatin. This combination therapy approach holds promise for improving treatment outcomes in lung cancer by potentially targeting multiple signaling pathways and enhancing the effectiveness of conventional chemotherapy. Further studies are needed to validate these findings and explore the underlying mechanisms of action (Qi et al.).

In the study conducted by Sahu et al. the researchers investigated the anti-diabetic properties of *Lyonia ovalifolia*, a plant predominantly found in the Indian Himalayas. The authors have discovered that extracts of the *L. ovalifolia* plant exhibit antiglycation activity and an increased level of GLUT4 translocation. Using LC-ESI-QTOF-MS/MS analysis, Sahu et al. confirmed the presence of 12 bioactive compounds in *L. ovalifolia*. Among these compounds, leucothol A, leucothol B, and rhodoterpenoids A were identified. Furthermore, Sahu et al. performed molecular docking analysis of the identified compounds with proteins involved in diabetes mellitus. This analysis led to the identification of potential drug candidates with promising interactions and therapeutic potential (Sahu et al.).

The final article, written by Hafeez et al. is a comprehensive review emphasizing the importance of Morusin, a natural product isolated from the plant *Morus alba*. The review explores various aspects of Morison, including its biological activities, pharmacological properties, and potential applications in different fields. Morison exhibits a wide range of biological properties, encompassing neuroprotective, antimicrobial, and antioxidant activities. Notably, it demonstrates significant anti-tumor properties and has shown efficacy against various types of cancer, including prostate, gastric, hepatocellular carcinoma, glioblastoma, breast, and pancreatic cancer. The diverse and potent effects of Morison make it a promising candidate for further research and potential therapeutic applications in cancer treatment (Hafeez et al.).

## Future prospective

The articles published in this Research Topic have highlighted numerous remarkable properties associated with the bioactive compounds derived from plants. These findings have laid a solid foundation for further research, which necessitates in-depth investigations into the specific bioactive compounds exhibiting anti-neurodegenerative, cytotoxic, ant proliferative, anti-tumor, and anti-diabetic properties. Such studies are essential for gaining a comprehensive understanding of the therapeutic potential and underlying mechanisms of action of these compounds. By delving deeper into their properties, researchers can uncover valuable insights that may pave the way for the development of novel treatments and interventions targeting neurodegenerative diseases, cancer, and diabetes (Alam et al., 2022; Huang et al., 2023). Indeed, while the plant extracts and various bioactive compounds reported in this Research Topic demonstrate efficacy against a broad range of diseases, the identification of the exact bioactive compounds becomes crucial for the targeted and effective treatment of specific diseases. Understanding the specific compounds responsible for the observed therapeutic effects allows researchers to focus their efforts on optimizing their isolation, synthesis, and formulation. This knowledge enables the development of more precise and tailored treatment strategies that can address the specific molecular targets and pathways involved in a particular disease. By identifying and characterizing these bioactive compounds, researchers can further enhance their potential as therapeutic agents and move closer to developing targeted treatments with improved efficacy and minimized side effects.

Indeed, future research focused on identifying the exact bioactive compounds present in the plant extract of *Ligusticum chuanxiong* Hort, which exhibits the ability to reduce *β*-amyloid peptide levels in *C. elegans*, holds great promise. By isolating and characterizing these bioactive compounds, researchers can gain a deeper understanding of their mechanisms of action and explore their potential as therapeutic agents for targeting *β*-amyloid-related pathologies, such as Alzheimer’s disease. Similarly, in the case of the plant extract of *L. ovalifolia*, which demonstrates antiglycation activity, the identification of the precise bioactive compound responsible for this property is of significant interest. By determining the specific compound with antiglycation properties, researchers can delve into its mode of action and investigate its potential as a novel therapeutic option for the treatment of diabetes and associated complications. This research may contribute to the discovery and development of new therapeutic approaches that target gyration processes, providing potential benefits for individuals affected by diabetes and related conditions.

Natural products (NPs) have a rich history of utilization in traditional medicine and have been derived from various sources such as plants, marine organisms, fungi, and other biological entities. The cutting-edge application of NPs, their unique molecular structures and modes of action represents a groundbreaking approach to enhancing human health and combating a wide range of diseases (Aventurado et al., 2020). By harnessing the power of NPs, researchers are exploring novel avenues for medical advancements and therapeutic interventions. These NPs offer a vast repertoire of bioactive compounds with diverse chemical properties and biological activities. The scientific community is actively engaged in developing innovative pharmaceuticals that specifically target disease pathways or biological targets. This endeavor involves the isolation and characterization of bioactive compounds. Moreover, advancements in biotechnological and synthetic biology techniques have paved the way for the controlled and scalable production of NPs. Genetic engineering, synthetic biology, and fermentation technologies play a crucial role in synthesizing or biosynthesizing compounds derived from NPs. These cutting-edge techniques enhance the accessibility of these compounds and help overcome challenges associated with sourcing these valuable resources. By harnessing these methodologies, researchers can revolutionize drug discovery and development, opening up new avenues for effective therapeutic interventions. These phytochemicals often exhibit specific mechanisms of action that enable them to interfere with critical pathways involved in cancer cell growth, proliferation, and survival (Newman and Cragg, 2020; Atanasov et al., 2021). By selectively targeting cancer cells, they can induce apoptosis (programmed cell death) or inhibit the uncontrolled cell division characteristic of cancer. The selective nature of these phytochemicals is advantageous in cancer therapy, as it reduces the risk of harming healthy cells and mitigates potential side effects associated with traditional chemotherapy drugs. This selectivity is attributed to the unique biochemical and genetic characteristics of cancer cells, which make them more vulnerable to the effects of these phytochemicals compared to normal cells.

The discovery and development of phytochemicals with cytotoxic and ant proliferative properties have thus opened up new avenues for developing targeted and personalized cancer treatments. Further research and clinical studies are underway to evaluate their efficacy, safety, and potential synergistic effects in combination with other therapeutic modalities. Overall, these phytochemicals hold great promise as lead compounds in the clinical management of cancer, offering a selective and specific approach to combat malignant cells while minimizing the impact on healthy tissues (Rai et al., 2022; Rai et al., 2023). The crude extract, displaying promising pharmacological activity, is subjected to bioactivity-guided fractionation. This involved employing various analytical techniques and subsequent phenotypic assays to isolate and identify the purified bioactive compounds (Atanasov et al., 2021). Nevertheless, this process encountered several challenges, including the presence of known NPs, NPs lacking drug-like properties, and the need for comprehensive NP characterization. To address these limitations, the development of techniques for dereliction, extraction, and pre-fractionation of extracts is crucial. Consequently, conducting extensive preclinical studies on NPs and extracts becomes essential, encompassing the evaluation of their safety profiles, efficacy in animal studies, therapeutic index, and potential regulatory mechanisms of action against targeted diseases. These comprehensive investigations hold promising prospects for future clinical implementation. Additionally, in light of the recent discoveries in this field, numerous other phytochemicals have been investigated for their potential in combating cancer (Newman and Cragg, 2020; Wang et al., 2023). The current emphasis of anticancer research should center on developing phytochemicals as leading-edge anticancer drugs exhibiting cytotoxic and anti-proliferative activity in an *in vitro* model along with anti-tumor and antiangiogenic activity in an *in vivo* model of various types of cancer. A particularly promising approach is combination therapy, which involves the strategic combination of two or more therapeutic agents. This synergistic approach has the potential to yield favorable results in the fight against cancer.
